# The Role of Inhaled Loxapine in the Treatment of Acute Agitation in Patients with Psychiatric Disorders: A Clinical Review

**DOI:** 10.3390/ijms18020349

**Published:** 2017-02-08

**Authors:** Domenico de Berardis, Michele Fornaro, Laura Orsolini, Felice Iasevoli, Carmine Tomasetti, Andrea de Bartolomeis, Nicola Serroni, Alessandro Valchera, Alessandro Carano, Federica Vellante, Stefano Marini, Monica Piersanti, Giampaolo Perna, Giovanni Martinotti, Massimo Di Giannantonio

**Affiliations:** 1 The National Health Service, Department of Mental Health, Psychiatric Service of Diagnosis and Treatment, Hospital “G. Mazzini”, ASL 4, 64100 Teramo, Italy; 2 Department of Neurosciences and Imaging, Chair of Psychiatry, University “G. D’Annunzio”, 66013 Chieti, Italy; nicola.serroni@aslteramo.it (N.S.); federica.vellante@gmail.com (F.V.); sfnmarini@gmail.com (S.M.); giovanni.martinotti@gmail.com (G.M.); digiannantonio@unich.it (M.D.G.); 3 New York Psychiatric Institute, Columbia University, New York, NY 10032, USA; dott.fornaro@gmail.com; 4 Polyedra Research Group Association, 64100 Teramo, Italy; laura.orsolini@hotmail.it (L.O.); a.valchera@ospedaliere.it (A.V.); 5 School of Life and Medical Sciences, University of Hertfordshire, Hatfield, Herts AL10 9AB, UK; 6 Villa S. Giuseppe Hospital, HermanasHospitalarias, 63100 Ascoli Piceno, Italy; 7 Laboratory of Molecular Psychiatry and Psychopharmacotherapeutics, Section of Psychiatry, Department of Neuroscience, University School of Medicine “Federico II”, 80133 Naples, Italy; felix_ias@hotmail.com (F.I.); carmine.tomasetti@aslteramo.it (C.T.); adebarto@unina.it (A.d.B.); 8 The National Health Service, Department of Mental Health, Psychiatric Service of Diagnosis and Treatment, Hospital “Madonna Del Soccorso”, 63074 San Benedetto del Tronto, Italy; alessandro.carano@gmail.com; 9 Hospital Pharmacy, Hospital “G. Mazzini”, ASL 4, 64100 Teramo, Italy; monica.piersanti@aslteramo.it; 10 Hermanas Hospitalarias, FoRiPsi, Department of Clinical Neurosciences, Villa San Benedetto Menni, Albese con Cassano, 22032 Como, Italy; pernagp@gmail.com; 11 Department of Psychiatry and Neuropsychology, University of Maastricht, 6200 MD Maastricht, The Netherlands; 12 Department of Psychiatry and Behavioral Sciences, Leonard Miller School of Medicine, University of Miami, Coral Gables, FL 33124, USA

**Keywords:** loxapine, inhaled, agitation, schizophrenia, bipolar disorder, antipsychotics, efficacy, tolerability

## Abstract

Loxapine is a first generation antipsychotic, belonging to the dibenzoxazepine class. Recently, loxapine has been reformulated at a lower dose, producing an inhaled powder that can be directly administered to the lungs to treat the agitation associated with psychiatric disorders, such as schizophrenia and bipolar disorder. Thus, the aim of this narrative and clinical mini-review was to evaluate the efficacy and tolerability of inhaled loxapine in the treatment of acute agitation in patients with psychiatric disorders. The efficacy of inhaled loxapine has been evaluated in one Phase II trial on patients with schizophrenia, and in two Phase III trials in patients with schizophrenia and bipolar disorder. Moreover, there are two published case series on patients with borderline personality disorder and dual diagnosis patients. Inhaled loxapine has proven to be effective and generally well tolerated when administered to agitated patients with schizophrenia and bipolar disorder. Two case series have suggested that inhaled loxapine may also be useful to treat agitation in patients with borderline personality disorder and with dual diagnosis, but further studies are needed to clarify this point. However, the administration of inhaled loxapine requires at least some kind of patient collaboration, and is not recommended in the treatment of severe agitation in totally uncooperative patients. Moreover, the drug-related risk of bronchospasm must always be kept in mind when planning to use inhaled loxapine, leading to a careful patient assessment prior to, and after, administration. Also, the higher costs of inhaled loxapine, when compared to oral and intramuscular medications, should be taken into account when selecting it for the treatment of agitation.

## 1. Introduction

Being faced with an agitated or violent patient is a challenge for every psychiatrist and associated health care professional, working in the everyday “real world” clinical practice [1]. Even if agitation or violence are not common symptoms of all psychiatric disorders, and the majority of persons with such disorders are not violent [2], it is undoubtable that these manifestations may more often occur in some psychiatric disorders (e.g., schizophrenia or bipolar disorders) [3,4], when there are comorbid conditions (e.g., substance and alcohol abuse or dependence, some personality disorders) [5], or lack of medication compliance [6].

The presence of psychotic symptoms has long been thought to be associated with agitation or violence, and this has often influenced the public opinion of associated illnesses, resulting in an increasingly perceived “stigma” of psychiatric disorders in general [7]. Nevertheless, the relationship between psychotic symptoms and agitation/violence, is very complex [8]. In fact, agitation and violence may be mediated by some clinical characteristics associated with schizophrenia or bipolar disorder (rather than schizophrenia or bipolar disorder per se), such as a heightened sensitivity to a perceived threat [9].

When considering the relationships between psychosis and agitation/violence, a pattern of personality traits related to psychosis (the so-called “threat/control-override”, TCO) [10,11], has received practical support as a likely explanatory variable for the psychosis-aggression association. As noted by Link and Stueve [10], only psychotic-like experiences that lead someone to fear a heightened perceived threat of harm (such as commanding auditory hallucinations, persecutory delusions, hypervigilance), while blocking internal constraints against violence (the “control-override” system), may precipitate agitation, aggression, and violent behaviours.

Furthermore, the agitation or violence associated with schizophrenia and mood disorders requires immediate treatment, to avoid injury to patients, nursing and medical staff, and others. The current guidelines for the management of severe agitation in schizophrenia and bipolar disorder recommend treatment with antipsychotic agents and/or BDZs, initiated as soon as possible after other conditions associated with agitation have been ruled out [11,12].

To date, there are some medications that are commonly used for the acute treatment of agitation/violence in psychiatric patients, and these include first-generation antipsychotics (FGAs), second-generation antipsychotics (SGAs), and benzodiazepines (BDZs) [13]. There are three possible routes for the administration of such drugs: oral, intramuscular (IM), or intravenous [11,12]. Even if FGAs have been commonly used in clinical practice for many years, because several SGAs (such as olanzapine, ziprasidone, and aripiprazole) are available in immediate-release IM preparations, and have been approved for the treatment of acute agitation in patients with schizophrenia and bipolar disorder, these are often the first-choice antipsychotics, together with BDZ, for the acute treatment of such patients [14] (Table 1).

Recently, a fourth route of administration (inhaled loxapine) has been made available for the treatment of agitation in psychiatric disorders [15]. In fact, loxapine, a FGA, has recently been reformulated at a lower dose, producing an inhaled powder that can be directly administered to the lungs [16,17].

Thus, the aim of this narrative and clinical review was to evaluate the efficacy and tolerability of inhaled loxapine in the treatment of acute agitation in patients with psychiatric disorders.

## 2. Overview of Inhaled Loxapine

### 2.1. Mechanism of Action

Loxapine is a FGA belonging to the pharmacological class of dibenzoxazepines, and it is structurally related to clozapine, differing from it in the position of one of its chlorine atoms, as well as the replacement of the diazepine group of clozapine, with the oxazepine group of loxapine [18,19]. Early studies identified a peculiar receptor profile for loxapine, which demonstrates a medium-to-strong affinity to dopamine D1, D2, and D4 receptors, as well as to serotonin 5HT2 receptors, with negligible affinity to glutamate NMDA receptors [20]. Although it has traditionally been considered a FGA, a significant number of studies have reported on the “atypical” pharmacodynamic properties of loxapine (similar to those of SGAs). Indeed, early in vitro reports showed that loxapine’s affinity to 5HT2 receptors seemed to be higher than its affinity to dopamine D2 receptors [21,22]. Moreover, the affinity to dopamine D4 receptors has also been reported as being higher than the affinity to D2 receptors, and it seems even higher than clozapine’s D4 affinity [20].

However, in vitro findings have not been confirmed by in vivo studies, in which loxapine demonstrated a clear lowering of the 5HT2/D2 affinity ratio [23]. This result could depend on the fact that loxapine is extensively metabolized in humans, to form hydroxylated metabolites, such as the 7-hydroxyloxapine, which has been found to have a 5-fold higher affinity to dopamine D2 receptors, when compared to the original compound [24]. A more recent study by Kapur et al. further demonstrated that loxapine equipotently blocks 5HT2 and D2 receptors in humans [25], thus invalidating the hypothesis that this drug might be similar to clozapine, which conversely displays a higher affinity and occupancy of 5HT2 receptors, when compared to D2 receptors [26]. Loxapine also has a high affinity to hystaminergic H1 and adrenergic α-1/α-2 receptors, and a moderate affinity to cholinergic M1 receptors, which are supposedly responsible for the sedation [27].

To strengthen the relationship between loxapine and FGAs, some studies have revealed that loxapine may largely increase the expression of D2 and D3 receptors’ mRNA, similarly to haloperidol, with the only exception of D1 receptors’ mRNA, which is induced to a greater extent by loxapine, thereby suggesting a propensity to induce extrapyramidal symptoms, similar to FGAs [28]. Moreover, loxapine has been reported to promote synaptic responses in the hippocampus at a similar level to haloperidol, whereas clozapine has no effect [29]. Clozapine, as is the case with other SGAs, may facilitate prefrontal cortical NMDA- and AMPA-mediated responses, whereas loxapine and haloperidol depress excitatory responses [30]. Characteristically, loxapine, together with other FGAs liable for inducing EPS (e.g., haloperidol, chlorpromazine, fluphenazine), has been found to induce the expression of the early gene *C-Fos*, specifically in the dorsolateral region of the caudate-putamen, which is implicated in the control of motor functions. In contrast, SGAs like clozapine peculiarly induce *Fos* reactivity in the prefrontal cortex, nucleus accumbens, and lateral septal nucleus, all implicated in the management of behaviour and emotions [31,32].

Preclinical behavioural studies have demonstrated that loxapine may induce catalepsy in rats to the same extent as olanzapine, and these effects may be abolished by clozapine administration, but only when it is administered after the catalepsy is fully developed [33]. In discriminative stimulus comparisons, loxapine may only induce minimal generalization, similarly to haloperidol, whereas clozapine has been demonstrated to induce full dose-related generalization in the absence of response suppression [34]. However, the close structural relationship between clozapine and loxapine could be responsible for the clozapine-like side effects of loxapine, such as weight gain and metabolic syndrome. Indeed, an in vitro study has demonstrated that loxapine may reduce intracellular glucose uptake by inhibiting the glucose transport in a way which is very similar to clozapine [35].

In contrast to the other FGAs, loxapine may promote dopamine release in the cortex and nucleus accumbens that is comparable with SGAs, an effect that denotes loxapine as having a unique profile, when compared to its closer companion drugs [36]. Puzzlingly, it has been demonstrated in preclinical studies that the isomer of loxapine, isoloxapine, possess “atypical” properties (similar to SGAs), when compared to its progenitor, putatively due to the 5HT2 and alpha2-oriented affinity to the isomer [37]. Isoloxapine, when compared to loxapine, behaves as a SGA, by inhibiting a conditioned avoidance response that does not induce catalepsy and hyperprolactinemia, but that does induce slight c-Fos expression in the dorsolateral striatum. All of these effects are ones which researchers ascribe to the very high occupancy of D2 receptors by loxapine, that invalidates whichever 5HT2-related “atypical” property could be displayed by the compound [38].

Brain distribution studies have reported that loxapine-hydroxylated metabolites (7-hydroxyloxapine), but not the progenitor, can be localized above all of the others in the striatum, demonstrating consistency with the D2 affinity of the compound [39]. Moreover, the intranasal administration of loxapine tends to induce less extrapyramidal symptoms (EPS) than oral administration, which is compatible with the higher levels of 7-hydroxyloxapine reached in the striatum after oral administration [40]. Last, in anaesthetized animals, intranasal loxapine reaches a higher bioavailability than in conscious specimens, due to the liver metabolism suppression which is caused by anaesthesia [41].

Loxapine is available in oral and intramuscular formulations. In 2012, the U.S. Food and Drug Administration (FDA) approved an inhalatory route of loxapine (Adasuve^®^) for the acute treatment of agitation associated with schizophrenia or bipolar I disorder in adult patients, with a 10 mg formulation (Table 1) [42,43]. However, inhaled loxapine is not approved for the treatment of patients with dementia-related psychosis and is contraindicated in patients with active airways disease [22].

### 2.2. Inhaled Loxapine: The Staccato^®^ System Delivery

The inhaled loxapine is administered through a hand-held, single-dosage, disposable breath-actuated tool (the Staccato^®^ system), specifically designed to quickly administer drug dry powder into the alveoli, with IV-like pharmacokinetics leading to a rapid systemic effect [19]. With the Staccato^®^ system, when the patient inhales through the device during a single normal breath, airflow is identified by a sensor that rapidly heats the loxapine-coated stainless steel heat source, vaporizing almost 90% of the loxapine stored in the device, usually in less than one second [44]. Then, the vapor cools quickly, condensing into >99.5% pure, excipient-free loxapine particles of 2 lm in diameter, suitable for alveolar deposition, which travel deep into the lungs through the inhaled airflow of the patient’s breath, ensuring a fast systemic absorption [45]. The entire process of drug delivery commonly happens in less than 1 s [24]. However, it is worth noting that some degree of patient co-operation is undoubtedly necessary [17].

### 2.3. Studies on Healthy Subjects and Pharmacokinetics of Inhaled Loxapine

Several studies on healthy volunteers have been conducted in order to assess the pharmacokinetics and tolerability of inhaled loxapine [46,47,48,49,50] (Table 2). The administration of inhaled loxapine results in a rapid absorption, with a Tmax of two minutes, reaching a mean maximum plasma concentration (*C*max) of 257 ± 219 ng/mL [26,29]. Loxapine is rapidly removed from the plasma and distributed throughout the body. A total of 96.6% of the loxapine is protein-bound. Loxapine is metabolized in the liver after oral administration, through several metabolic pathways [22] (hydroxylation, *N*-oxidation, and de-methylation form 8-OH-loxapine [CYP1A2] and 7-OH-loxapine [CYP3A4/CYP2D6], loxapine *N*-oxide by flavonoid monoamine oxidases and amoxapine, respectively) [51]. Metabolic interactions should be minimal, due to extensive metabolism through various pathways. The conjugated metabolites of loxapine are eliminated through the kidneys and, to a lesser extent, unconjugated via the faeces. The drug’s half-life ranges from six to eight hours. No effect on cardiac repolarization, as measured by the QTc interval, emerged during the two studies on healthy subjects [27,28].

### 2.4. Clinical Trials of Inhaled Loxapine in the Treatment of Agitation in Psychiatric Disorders

The efficacy of inhaled loxapine has been evaluated in one Phase II trial and in two Phase III trials. Moreover, there are two published case series (Table 3).

Concerning psychiatric disorders, in a phase II randomized, double-blind, placebo-controlled study, Allen et al. [52] evaluated, both before and after 2 h, 129 agitated patients with schizophrenia or schizoaffective disorder, who were randomized to receive a single inhalation of 5 or 10 mg of loxapine or a placebo, in a clinical or hospital setting. The inhaled loxapine produced a rapid improvement in the agitated patients, and statistically significant differences in efficacy were found for the 10-mg dose, when compared with the placebo, with results suggesting that 5 mg may also be effective. Lesem et al. [53] conducted a phase III, randomised, double-blind, placebo-controlled, parallel-group study on 344 agitated patients with schizophrenia, who were administered two or three doses of inhaled loxapine (5 or 10 mg), or a placebo. They found that the inhaled loxapine was an effective treatment for agitation in schizophrenia, and that both the 5 and 10 mg doses resulted in significantly larger decreases in the Positive and Negative Syndrome Scale–Excited Component (PANSS–EC), during the 2 h period after the first dose. The last phase III, randomized, double blind, placebo-controlled, parallel group in the patient trial was conducted by Kwentus et al. [54], who evaluated 314 agitated patients with bipolar I disorder over 24 h (with manic or mixed episodes), randomizing them (1:1:1) for the inhalation of loxapine 5, 10 mg, or a placebo. The administration of both doses of inhaled loxapine significantly reduced agitation, when compared with the placebo, as was reflected in the PANSS-EC score, and this was manifested 10 min after dose 1, with both doses.

Moreover, two open label case series, on five agitated patients with borderline personality disorder (BPD) [55] and 14 agitated patients with dual diagnosis [56], showed that inhaled loxapine 10 mg was rapid, effective, and well-accepted in both groups, without adverse effects (AEs).

### 2.5. Common Adverse Effects of Inhaled Loxapine in Patients with Psychiatric Disorders

Overall, in the Phase II trial and in two Phase III trials. As well as in the case series, inhaled loxapine was well-tolerated in patients with schizophrenia or bipolar disorder, with no excessive sedation. The majority of AEs were mild to moderate in intensity and did not require intervention: the most common AEs in patients receiving inhaled loxapine were dysgeusia, throat irritation, and sedation [23,24,25]. As reported by Citrome [57], the number needed to harm (NNH) for dysgeusia, for inhaled loxapine versus the placebo, was 16 (95% CI 10–58) for the 5 mg dose and 11 (95% CI 7–23) for the 10 mg dose, whereas the NNH for throat irritation was only statistically significant for the 10 mg dose (44; 95% CI 23–472). Moreover, the NNH for sedation/somnolence was not statistically significant for either the 5- or 10-mg dose of inhaled loxapine.

Concerning severe adverse effects (AEs), in the study of Allen et al. [24], no patients withdrew from the study due to AE. Only one episode of dystonic reaction was observed (jaw clenching), in a patient with a history of jaw clenching, secondary to antipsychotics. Interestingly, three serious adverse events, including one death, were reported as occurring at least six days after the administration of loxapine, but none were judged by the investigators to be related to treatment with loxapine. In the trial of Kwentus et al. [26], there was one severe AE in a loxapine-treated patient (sedation in a 10 mg patient), but inhaled loxapine was generally well-tolerated, and most AEs were judged to be of mild or moderate severity and were resolved without intervention. Moreover, one patient (5 mg group) experienced moderate akathisia, which was judged to probably be treatment-related, and which was resolved after benztropine treatment. Lesem et al. [25] reported severe AEs for three patients in the 10 mg group. In the 10 mg group, one patient developed neck dystonia and oculogyration that were judged treatment-related, and which required benztropine to be resolved. One patient showed a severe treatment-related sedation and one developed severe infectious gastroenteritis that was judged to be unrelated to treatment, requiring hospitalisation before being resolved.

No effects on the cardiovascular system (i.e., QTc prolongation or torsades de pointes) were observed with inhaled loxapine in all of the studies and this finding is in line with trials on healthy volunteers [20,21,22].

### 2.6. Pulmonary AEs of Inhaled Loxapine

As loxapine has been reformulated for direct inhalation into the lungs, where it enters the alveoli for rapid access to the arterial circulation, some concerns relating to its safety have been raised, regarding the onset of potential AEs, in the form of asthma, wheezing, and bronchospasms. In fact, patients with clinically significant acute or chronic pulmonary diseases were excluded from the phase II and III clinical trials. Interestingly, pulmonary AEs in all of the reviewed studies were rare and were mild to moderate, without severe complications or death. Wheezing and bronchospasms were reported in one randomized study [24] and required intervention (one patient resolved these AEs with albuterol, two puffs by a metered-dose inhaler).

Moreover, Gross et al. [58] conducted two separate, randomized, double-blind, parallel-arm, placebo-controlled trials, comparing two administrations of inhaled loxapine (10 mg) and a placebo, 10 h apart, in 52 subjects with asthma and in 53 subjects with chronic obstructive pulmonary disease (COPD). Spirometry results, including the forced expiratory volume in 1 s (FEV1), the forced vital capacity (FVC), and FEV1/FVC with FEV1, were considered the primary outcome measures. Spirometry tests were performed in the hour before the first dose of the study’s treatment, and at 0.25, 0.5, 1, 2, 4, 6, 10, 10.25, 10.5, 11, 12, 14, 16, 24, and 34 h after that dose. The results showed that, in subjects with asthma and COPD, inhaled loxapine may cause an airway effect (FEV1 decline and bronchospasm) that is commonly reversible with a short-acting β-agonist bronchodilator (such as albuterol). However, the authors recommended that a brief pulmonary assessment (i.e., history and screening physical examination) should be performed, to select appropriate patients for undergoing treatment with inhaled loxapine. Moreover, they point out that a short-acting β-agonist bronchodilator should be readily available in real-world medical or psychiatric emergency settings, when choosing inhaled loxapine for the treatment of agitation.

## 3. A Clinical Perspective on the Role of Inhaled Loxapine in the Anti-Agitation Drugs Armamentarium: Pros and Cons

The formulation of inhaled loxapine may be a useful pharmacological tool in the treatment of acute agitation associated with psychiatric disorders [17,31,32,33]. In fact, inhaled loxapine quickly reduces acute agitation in patients with schizophrenia or bipolar disorder, through a non-invasive route of administration with an onset of the effect within ten minutes [59]. Moreover, inhaled loxapine is generally well-tolerated, and the most common AEs are dysgeusia, throat irritation, and mild sedation [48,52,53,54], that do not usually require intervention.

Inhaled loxapine may have some advantages when compared to other drugs that are widely employed to treat acute agitation [60]. As patient preference is an issue that should always be considered in the choice of treatment of psychiatric disorders, especially during a crisis (such as an episode of agitation associated with schizophrenia or bipolar disorder), in order to establish a valid and healthy therapeutic relationship [61], the patient’s preference for treatment delivery options has been demonstrated. It revealed that inhalation was associated with a significant utility gain, when compared to injections or tablets, for controlling agitation episodes, and this may be particularly true for inhaled loxapine [62]. Moreover, the delivery of inhaled loxapine directly into the lung has several potential advantages. These include a rapid onset of the anti-agitation effect (within a couple of minutes, generally 10), and a less invasive and needle-free systemic delivery of drugs [48,52,53,54,55,56]. Furthermore, the inhaled formulation of loxapine avoids transit through the gastrointestinal system and, therefore, the hepatic first-pass metabolism achieves a bioavailability of 9.1 mg of the 10 mg dose [25].

Another great advantage of inhaled loxapine is the lack of the effect on cardiac repolarization. In fact, Cassella et al. [48] showed that two therapeutic doses of inhaled loxapine did not cause threshold QTc prolongation, thus confirming the previous results of Spyker et al. [47], who demonstrated that there was no apparent QT prolongation associated with the therapeutic dose of inhaled loxapine. Also, in clinical studies of agitated patients with psychiatric disorders, inhaled loxapine did not cause QTc prolongation or torsade de pointes [49,52,53,54,55].

However, there are some disadvantages that should be considered. Even if the delivery system is innovative for an antipsychotic drug, it presumes that some kind of patient collaboration is undoubtedly required [16,48,52,53,54,55,56,57]. This may be true for most cases of mild-to-moderate agitation, and also for some cases of severe agitation. Therefore, the use of inhaled loxapine is not the first-line treatment for severe agitation in totally uncooperative patients, and IM medication should be used.

Although the majority of users of mental health services are not uncooperative, it is clear that in an everyday clinical practice, a small yet significant minority is non-collaborative, in both inpatient settings and emergency services [63]. This may limit the usage of inhaled loxapine in most cases of severe agitation, even if, when using non-pharmacological strategies such as verbal de-escalation, patient collaboration may finally be obtained [64]. Moreover, in the severe agitation of totally uncooperative patients, an IM medication should be used when the patients are not willing to accept a medication that they need to take orally [63]. Inhaled loxapine should only be used in the case of agitated, but somewhat or partial collaborative patients, and in such cases, this drug may be as useful as IM SGAs, to obtain a more rapid tranquillisation than the oral administration [59]. Moreover, it frequently occurs that, in everyday clinical practice, the agitated patients may initially and apparently accept the oral medications (often to avoid involuntary admissions), but they may spit out the oral medications, or need a relatively long time to achieve tranquilisation after oral administration [1,2,3]. The use of inhaled loxapine may overcome these shortcomings, as the administration and the rapid onset of the anti-agitation effect are warranted [52,53,54,55].

However, the major concern is the drug-related risk of bronchospasm, which can lead to respiratory distress and respiratory arrest [65]. It is worth noting that inhaled loxapine is contraindicated in patients with a current diagnosis or history of asthma, COPD, or other lung diseases associated with bronchospasms, or those with acute respiratory symptoms or signs (such as wheezing) [22]. It is also contraindicated in patients taking medications used to treat airway diseases and those with a history of bronchospasms following inhaled loxapine treatment in the past [22]. Inhaled loxapine has a “Boxed Warning” and is only available through a restricted program under a Risk Evaluation and Mitigation Strategy (REMS), called “ADASUVE REMS” [66]. Health care facilities using inhaled loxapine are required to have immediate, on-site access to equipment and personnel trained to manage acute bronchospasms, including advanced airway management (i.e., intubation and mechanical ventilation). These facilities must have a short-acting bronchodilator (e.g., albuterol), including a nebulizer and inhalation solution, for the immediate treatment of bronchospasms [67]. When choosing inhaled loxapine to treat agitation in psychiatric disorders, patients must be screened for a history or symptoms of asthma, COPD, or other pulmonary diseases, and monitored for signs or symptoms of bronchospasms following treatment [68].

Another concern may be the costs of inhaled loxapine. In the United States, the costs are relatively higher than those of the oral and IM medication currently employed to treat agitation in psychiatric disorders, and this should also be considered and taken into account when choosing inhaled loxapine [69].

## 4. Conclusions

**Figure 1:**
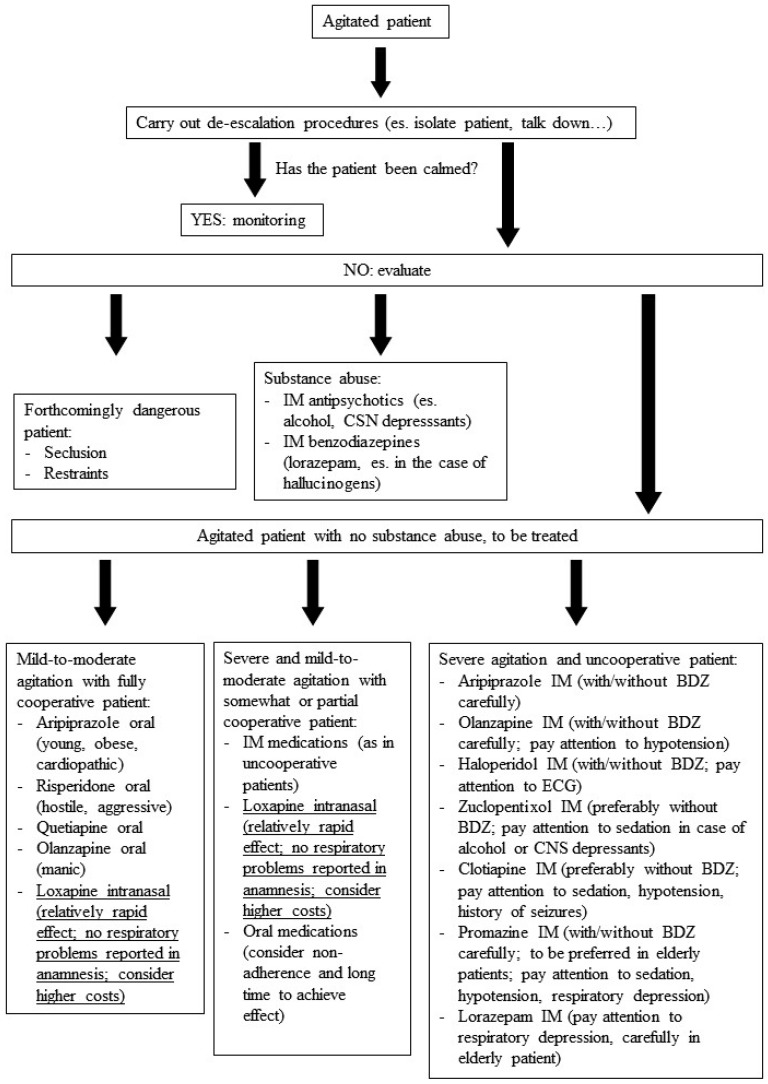
Management of acute agitation: the suggested position of intranasal FGA loxapine (adapted and modified from Schleifer [70].

In sum, it is always a good thing to have another drug for the treatment of agitation in psychiatric disorders, with an innovative delivery system. Inhaled loxapine has proven to be effective and generally well-tolerated when administered to agitated patients with schizophrenia and bipolar disorder, with a relatively rapid onset of action. However, even if the delivery system is innovative for an antipsychotic drug, it presumes that some kind of patient collaboration is undoubtedly required. Two case series have suggested that inhaled loxapine may also be useful to treat agitation in patients with BPD and with dual diagnosis, but further studies are needed to support this. However, the administration of inhaled loxapine requires some kind or full patient collaboration, which may be achieved in most cases using verbal de-escalation techniques. Moreover, the drug-related risk of bronchospasm must always be kept in mind when planning to use inhaled loxapine, leading to a careful patient assessment prior to administration. Also, the higher costs of this innovative formulation should be taken into account when choosing inhaled loxapine for the treatment of agitation. Moreover, the use of inhaled loxapine is not recommended as a first-line treatment for severe agitation in totally uncooperative patients. On the basis of the present review, the proposed position of inhaled loxapine in the treatment of acute agitation is described in Figure 1 (adapted and modified from Schleifer [70]).

## Figures and Tables

**Table 1:** Characteristics of inhaled First Generation Antipsychotic (FGA) loxapine.

Route of Administration	Inhalatory
Half Life	6–8 h
Time to peak concentration	2 min
Preparation instructions	Inhalation powder. 10 mg unit in a single-use inhaler
Food and Drug Administration (FDA) Indications	Acute treatment of agitation associated with schizophrenia or bipolar I disorder in adults
Available Dosage	10 mg
Maximum Dosage	10 mg/24 h
Average dose for tranquillization	10 mg/24 h
Recommendations	Administration requires a somewhat, partial or fully cooperative patient. Contraindicated in patients with diagnosis or history of asthma, Chronic Obstructive Pulmonary Disease (COPD), other lung disease associated with bronchospasm or in patients with acute respiratory symptoms or signs and with current use of medications to treat airways disease. It is not approved for the treatment of patients with dementia-related psychosis
Costs	$174/dose in USA (EUR price not yet available)

**Table 2:** Published studies on inhaled FGA loxapine in healthy subjects.

Authors	Year	Study Sample	Study Design	Number of Subject	Study Aims	Dose (mg/Day)	Main Findings
Spyker et al.	2010	Healthy subjects	Randomized, double-blind, placebo-controlled, dose escalation study	50	To determine the pharmacokinetic characteristics, safety, and tolerability of single doses of inhaled loxapine	0.625, 1.25, 2.5, 5.0, or 10 mg of loxapine or placebo	The inhalation of loxapine represented a safe and well-tolerated route for rapidly achieving therapeutic plasma concentrations
Spyker et al.	2014	Healthy subjects	randomized, placebo-controlled, double-blind crossover study	48	To evaluate effects of inhaled loxapine on the QTc interval	Inhaled loxapine 10 mg or inhaled placebo	No QTc prolongation was observed with the therapeutic dose of inhaled loxapine
Cassella et al.	2015	Healthy subjects	randomized, double-blind, active- and placebo-controlled, crossover, thorough QT study	60	To assessed the effect of two inhaled loxapine doses on cardiac repolarization as measured by QTc interval	inhaled loxapine (10 mg) 2 h apart+oral placebo, two doses of inhaled placebo+oral placebo, or two doses of inhaled placebo+oralmoxifloxacin (400 mg; positive control)	The two therapeutic doses of inhaled loxapine did not cause threshold QTc prolongation
Spyker et al.	2015	Healthy subjects	Randomized, double-blind, crossover study	22	Pharmacodynamic effects and safety of single-dose inhaled loxapine and IM lorazepam in combination versus each agent alone	lorazepam 1 mg IM, inhaled loxapine 10 mg, placebo IM (alone or combined)	Concomitant administration of inhaled loxapine and lorazepam in healthy volunteers showed no difference in its effect on respiration rate or pulse oximetry (primary endpoints) versus either drug alone

**Table 3:** Published studies on inhaled FGA loxapine in the treatment of agitation in psychiatric disorders.

Authors	Year	Study Sample	Study Design	Number of Subject	Study Aims	Dose (mg/Day)	Main Findings
Allen et al.	2011	Agitated patients with schizophrenia	Phase II, randomized, double-blind, placebo-controlled study	129	To evaluate inhaled loxapine for acute treatment of agitation in schizophrenia	Single inhalation of 5 or 10 mg of loxapine or placebo	Inhaled loxapine was generally safe and well tolerated and produced rapid improvement in agitated patients with psychotic disorders
Lesem et al.	2011	Agitated patients with schizophrenia	Phase III, randomised, double-blind, placebo-controlled, parallel-group study	344	To evaluate inhaled loxapine for acute treatment of agitation in schizophrenia	Two or three doses of inhaled loxapine (5 or 10 mg) or placebo	Inhaled loxapine was a well-tolerated and effective treatment of agitation in schizophrenia
Kwentus et al.	2012	Agitated patients with bipolar I disorder	Phase III, randomized, double blind, placebo-controlled, parallel group inpatient study	314	To evaluate inhaled loxapine for the acute treatment of agitation in patients with bipolar I disorder	Inhaled loxapine 5 mg or 10 mg or inhaled placebo	Inhaled loxapine provided a rapid, non-injection, well-tolerated acute treatment for agitation in patients with bipolar I disorder
Krüger et al.	2015	Agitated patients with borderline personality disorder	Case series	5	To evaluate inhaled loxapine for emergency treatment of agitated patients with borderline personality disorder	Inhaled loxapine 10 mg	Inhaled loxapine was safe, well tolerated and produced rapid improvement in agitated patients with borderline personality disorder
Roncero et al.	2016	Patients with dual diagnosis	Retrospective case Series	14	Efficacy of inhaled loxapine on episodes of psychotic agitation in patients with dual diagnosis	Inhaled loxapine 10 mg	Inhaled loxapine was rapid, effective, and well accepted in all dual-diagnosis patients presenting with acute agitation in the emergency setting
